# Genomic Diversity and Introgression in *O. sativa* Reveal the Impact of Domestication and Breeding on the Rice Genome

**DOI:** 10.1371/journal.pone.0010780

**Published:** 2010-05-24

**Authors:** Keyan Zhao, Mark Wright, Jennifer Kimball, Georgia Eizenga, Anna McClung, Michael Kovach, Wricha Tyagi, Md. Liakat Ali, Chih-Wei Tung, Andy Reynolds, Carlos D. Bustamante, Susan R. McCouch

**Affiliations:** 1 Department of Biological Statistics and Computational Biology, Cornell University, Ithaca, New York, United States of America; 2 Department of Genetics, Stanford University, Stanford, California, United States of America; 3 Department of Plant Breeding and Genetics, Cornell University, Ithaca, New York, United States of America; 4 Dale Bumpers National Rice Research Center, Agricultural Research Service (ARS), United States Department of Agriculture (USDA), Stuttgart, Arkansas, United States of America; 5 Rice Research and Extension Center, University of Arkansas, Stuttgart, Arkansas, United States of America; University of Texas Arlington, United States of America

## Abstract

**Background:**

The domestication of Asian rice (*Oryza sativa*) was a complex process punctuated by episodes of introgressive hybridization among and between subpopulations. Deep genetic divergence between the two main varietal groups (*Indica* and *Japonica*) suggests domestication from at least two distinct wild populations. However, genetic uniformity surrounding key domestication genes across divergent subpopulations suggests cultural exchange of genetic material among ancient farmers.

**Methodology/Principal Findings:**

In this study, we utilize a novel 1,536 SNP panel genotyped across 395 diverse accessions of *O. sativa* to study genome-wide patterns of polymorphism, to characterize population structure, and to infer the introgression history of domesticated Asian rice. Our population structure analyses support the existence of five major subpopulations (*indica*, *aus*, *tropical japonica*, *temperate japonica* and *GroupV*) consistent with previous analyses. Our introgression analysis shows that most accessions exhibit some degree of admixture, with many individuals within a population sharing the same introgressed segment due to artificial selection. Admixture mapping and association analysis of amylose content and grain length illustrate the potential for dissecting the genetic basis of complex traits in domesticated plant populations.

**Conclusions/Significance:**

Genes in these regions control a myriad of traits including plant stature, blast resistance, and amylose content. These analyses highlight the power of population genomics in agricultural systems to identify functionally important regions of the genome and to decipher the role of human-directed breeding in refashioning the genomes of a domesticated species.

## Introduction

Asian rice (*O. sativa*) has been cultivated for an estimated 10,000 years [1] and currently feeds more than one third of the world's population. As a key food staple, its management and genetic improvement is critical to human health and well-being, and understanding its population structure and domestication history is directly relevant to the design of more efficient and productive plant improvement programs. Rice also serves as an excellent model system for studying plant evolutionary genomics due to the broad range of morphological, physiological and developmental diversity found in both *O. sativa* and its widely distributed wild ancestor, *O. rufipogon/O. nivara*.

Rice varieties are traditionally classified into two major subspecies or varietal groups, *Indica* and *Japonica*, which differ in their adaptation to different climatic, ecogeographic and cultural conditions [2]. *Indica* varieties are widely grown in lowland tropical areas throughout South and Southeast (SE) Asia and China, while *Japonica* varieties are cultivated in both lowland and high-elevation upland areas of tropical SE Asia, and in colder, temperate climates, including northeastern Asia, Europe, western US, Chile and Australia [3]. The two varietal groups exhibit broadly overlapping phenotypic distributions for many morphological traits, though differences in grain shape, phenol reaction, amylose content and tillering ability are traditionally used to distinguish them. Rice cultivars within each varietal group are differentiated genetically across a host of molecular marker types including isozymes [4], [5], RFLPs [6], [7], SSRs [8], [9], SNPs [10]–[12] and Tranposon Insertion Polymorphisms [13] with increasing resolution of population structure with increasing marker density and informativeness. The most comprehensive studies of genetic variation in terms of number of accessions genotyped [5], [9], [14] and number of markers scored [11], [12] concur that domesticated Asian rice land races can be broadly classified into five subpopulations: *indica*, *aus*, *temperate japonica*, *tropical japonica* and *aromatic* (hereafter referred to as *Group V*, based on isozyme classification [5]). Of these, *indica* and *aus* cluster within the *Indica* varietal group, while *temperate japonica*, *tropical japonica* and *Group V (aromatic)* varieties cluster within the *Japonica* varietal group [9]. Two smaller subpopulation groups identified by Glaszmann [5], *ashwina* (Isozyme Group III) and *rayada* (Isozyme Group IV) frequently go undetected, often because they are under-represented in rice diversity studies.

These genetically defined subpopulations exhibit substantial genetic divergence as measured by a host of statistics including Wright's *F_ST_* [15], Bayesian clustering algorithms such as InStruct [16] and STRUCTURE [17], [18] and coalescent-based parametric population structure models [12]. However, there is clear evidence that gene flow between the subpopulations and even back into the wild rice [19] has resulted in the introgression of genomic segments over the course of the domestication process and, most recently, as part of concerted plant breeding programs. While we do not know the extent of introgression across the genome, several studies have identified specific regions carrying important agronomic traits such as shattering ability, pericarp color, seed length, seed number, fragrance, plant height and tiller angle that are identical-by-descent across divergent germplasm [20]–[28].

Recent years have seen great advances in utilizing association mapping as a genomics tool in diverse plant species, including Arabidopsis, maize, barley and rice. Arabidopsis is in the lead as far as the SNP genome coverage in association studies [29], , although the size of the mapping population is still relatively small. Association studies in maize have been largely carried out using a Nested Association Mapping (NAM) population [31], [32]. Although this population has good power and advantages, genetic diversity is limited by the small number of initial founder lines used to create the NAM population. Because the barley genome is not yet sequenced, the most recent association study utilizing 1524 unigene SNPs [33], [34] was limited by the resolution of the genetic linkage map and identifying candidate genes nearby depended on homology searches against other species. Most association studies in rice to date have used small numbers of RFLP and SSR markers [35]–[37]. Our new SNP array provides an opportunity to test the feasibility of genome-wide SNP-based association studies in a diverse panel of *O. sativa* germplasm.

The main goal of this study was to perform a genome-wide assessment of introgression among the subpopulations of *O. sativa* and to explore the feasibility of genome-wide association and admixture mapping in domesticated Asian rice. To address this objective, we designed an Illumina GoldenGate SNP assay from a high quality subset of the SNPs discovered in 20 diverse *O. sativa* landraces by the *Oryza*SNP project [11]. Using our 1,536 Illumina GoldenGate assay, we genotyped a panel of 395 diverse landrace and elite varieties of *O. sativa* and used the data to infer population structure, determine the extent of admixture, and document regions with significant degrees of introgression.

## Results

### Population structure and genetic relationships

**Figure 1:**
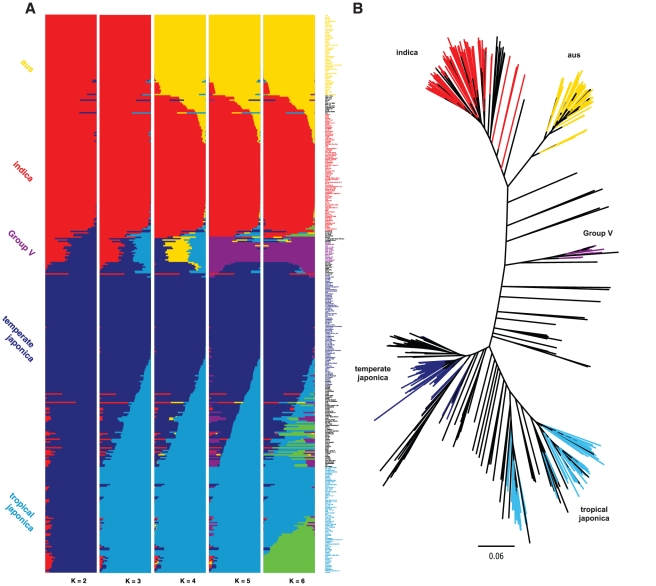
Population structure in *O. sativa* estimated from the GoldenGate SNP set. (A) Population structure estimate from STRUCTURE output for K = 2 to K = 6. (B) Phylogenetic tree. The branch tips of the tree are colored according to the subpopulation assignment in (A) when K = 5.

The 1536 SNPs included in the GoldenGate assay developed for this study represent approximately 1% of the SNP discovery pool [11] and were selected to provide 1 SNP approximately every ∼260 kb across the 12 chromosomes of rice (see Materials and Methods). From the original 1,536 SNPs on the array, 1,311 had high quality scores and were used to cluster genotypes having >1% minor allele frequency in our dataset. To analyze the population structure of the 395 *O. sativa* accessions, we performed a Bayesian clustering analysis using STRUCTURE with varying levels of *K* (details in Materials and Methods section). Specifically, at *K* = 2, we separated the two main varietal groups, *Indica* and *Japonica* (Figure 1A). When *K* was increased from three to five, each new cluster corresponded to one of the five main subpopulations - *indica*, *aus*, *temperate japonica*, *tropical japonica* and *Group V*. The *tropical* and *temperate japonica* groups diverged at *K* = 3, while the *aus* subpopulation emerged as an independent group at *K* = 4, and the *Group V* group emerged at *K* = 5. At *K* = 6, a subgroup of 20 *tropical japonica* varieties, representing germplasm from US breeding programs, was genetically distinguishable from the rest of that subpopulation. This highlighted the unique ancestry and breeding history of US *tropical japonica* varieties [38]. Clusters identified by increasing *K* beyond six did not contain a single individual with a majority of ancestry in the new cluster, indicating that for the number of markers evaluated here, *K*>6 does not improve population structure resolution. We also evaluated genetic relationships among the accessions by generating a neighbor-joining population tree based on pairwise allele-sharing distances. This analysis supported the same groupings as the Bayesian cluster analysis (Figure 1B).

While most of the accessions were classified into one of the five groups at *K* = 5, we classified 90 accessions as admixtures because they showed less than 80% of estimated ancestry derived from any single subpopulation (Figure 1A). The majority of the admixture occurs within the *Indica* or *Japonica* varietal group, with a few notable exceptions such as cv 923 from Madagascar, with ancestry from *indica*, *tropical japonica* and *aus*; Pirinae 69 from the former Yugoslavia and C1-6-5-3 from Mexico, both having ancestry from *indica* and *temperate japonica*, and K65 from Suriname with ancestry from *tropical japonica*, *indica*, *Group V* and *aus* (Supplemental Table S1). When the proportion of ancestry required to identify subpopulation identity was reduced to 60%, only 43 accessions could not be clustered clearly within a single subpopulation and were classified as admixed.

### Between group differences

To explore the genomic distribution of subpopulation differences, we examined *F_ST_* values between pairs of subpopulation groups defined at K = 5. *F_ST_* values ranged from 0.23 to 0.53 (Table 1). The lowest *F_ST_* group pair was observed between *indica* and *aus*, while the highest *F_ST_* values were found between *indica* or *aus* and *temperate japonica*. These results were generally consistent with estimates derived from far fewer SSRs [9], except that our SNP assay more clearly differentiated the *tropical* and *temperate japonica* groups.

**Table 1:** Fst between pairwise subpopulation groups.

	*aus*	*indica*	*temperate japonica*	*tropical japonica*
*Group V*	0.43	0.40	0.42	0.33
*aus*		0.23	0.53	0.43
*indica*			0.52	0.41
*temperate japonica*				0.35

When the common set of 1,311 SNPs shared by the *Oryza*SNP dataset (20 varieties) and the current study (395 varieties) was used to compute *F_ST_* values between the *Indica (indica)* and *Japonica (temperate japonica* and *tropical japonica)* varietal groups, significant differences were observed in the estimates, mainly due to the dramatically different sample sizes. We reasoned that the low number of SNPs in our GoldenGate assay, coupled with our SNP selection regime (see Materials and Methods), could bias regional patterns of variation along the genome. To address this question, we computed *F_ST_* estimates along the chromosomes from the 159,879 SNPs in the original SNP discovery pool (*Oryza*SNP dataset) using a 100kb sliding window and compared results from the GoldenGate assay with the denser set of *Oryza*SNP data (Supplemental Figure S1). We determined that the true pattern of polymorphism would be evident where the pattern of the two estimates agreed. Using this approach, we discovered a large region (∼3 Mb) of unusually low divergence between *Indica* and *Japonica* near 10Mb on chromosome 5, extending through the centromere. The region was also reported in [39], which is characterized by a high frequency of repetitive DNA (based on annotation in the Nipponbare genome (http://rice.plantbiology.msu.edu/) and it has low SNP polymorphism in all subpopulations except *aus*. The cause of the large sweep of low polymorphism in four of the subpopulations is intriguing and raises interesting questions about its possible functional significance, as well as about the origin of the many unique alleles found in the *aus* subpopulation.

### Inter-subpopulation introgression

**Figure 2:**
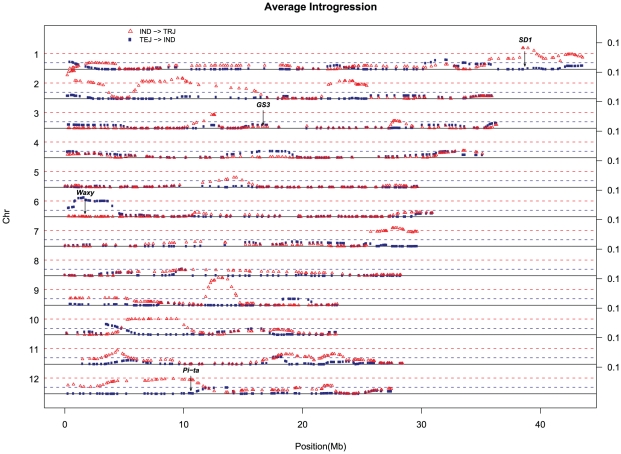
Average Introgression component in subpopulations. Each panel represents one chromosome and each SNP is represented as one point in the figure. Here we illustrate introgression from *indica (IND*) into *tropical japonica* (*TRJ*) (red) and from *temperate japonica (TEJ)* into *indica (IND)* (dark blue). The dashed lines are the 5^th^ percentile of all the SNPs for each introgression with the same color. Four genes *SD1*, *GS3*, *Waxy*, *Pi-ta* located in or near peak regions are indicated along the genome.

Although we observe deep divergence between the different rice subpopulations, rice varieties are distinguished by a significant degree of admixture. To characterize the source and extent of introgressions in the genomes of diverse varieties, we used Bayesian STRUCTURE analysis (K = 5) to quantify the degree of admixture in different regions of the genome (Figure 2). Because there was only one *Group V* used in the initial SNP discovery and only a small number of *Group V* accessions was included in this study (n = 14), we were unable to obtain reliable results for this subpopulation, and focus instead on documenting admixture between *indica*, *tropical japonica*, and *temperate japonica*. Using the subpopulation identity defined by the 1311 SNPs of population structure analysis above, most of the accessions show very little evidence of introgression from other groups. However, there is significantly more *indica* introgression into *tropical japonica* than into *temperate japonica* (one-sided t-test p-value = 0.0003 comparing the average introgression in each individual in the two subgroups), as shown in Supplemental Figure S2. The top fifth-percentile of average introgression for all accessions in each subpopulation are all less than 0.06 (Figure 2). Yet some regions in the genome show significantly more introgression than background (more than the top fifth-percentile). For example, we detect evidence of introgression from *indica* into *tropical japonica* on chromosomes 1, 2, 7, 9 and 12, while the region most commonly associated with an introgression from *temperate japonica* into *indica* is at the top of the short arm of chromosome 6. Interestingly, many of the larger regions of introgression contain genes of agronomic importance known to be the targets of artificial selection. Below, we provide details about a few key genes that lie within these regions.

### *sd1*: a recessive allele conferring semi-dwarf stature

**Figure 3:**
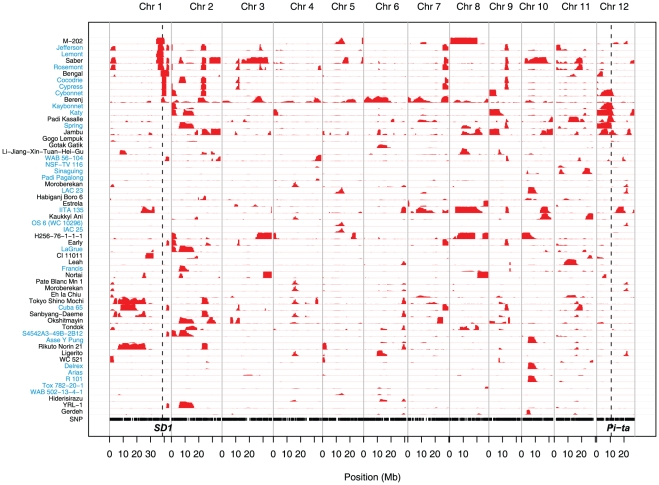
Regions of introgression from *indica* into *tropical japonica*. SNP positions are shown as black vertical bars across the bottom; vertical grey lines indicate chromosomes; horizontal grey lines indicate accessions; introgressed regions (defined by ≥5 SNPs) shown in red. The height of the red regions corresponds to the probability of introgression, with a maximum value of 1. Only *tropical japonica* and *admixed* accessions (where *indica* component is less than 25%) are plotted in the figure. *SD1* and *Pi-ta* positions shown as vertical dashed lines. Accession IDs are colored in light blue for *TRJ* and black for admixed accessions.

The gene *SD1* (*OsGA20 oxidase*) is a key determinant of plant stature and played a key role during the Green Revolution [40], [41]. Located at 38.7 Mb on rice chromosome 1, it is part of the gibberellic acid pathway. The recessive allele, *sd1*, confers semi-dwarf stature and contributed to massive yield improvements throughout most of Asia by increasing the harvest index and helping to prevent lodging [42]. The trait was first identified in an *indica* variety from Taiwan, Deo Gee Woo Gen (DGWG), and the DGWG allele has been widely used over the last 50 years to enhance the productivity of both *Indica* and *Japonica* varieties [43]. Using the GoldenGate assay, we document that there is highly elevated introgression from *indica* into many *tropical japonica* varieties near the *SD1* gene (Figure 2) in agreement with the historical record (Figure 3) [44]. These varieties include Cypress [45], Lemont [46], Cocodrie [45], Cybonnet [47], Rosemont [48], Jefferson [49], all of which are known to be semi-dwarf plants. Four semi-dwarf admixed accessions, including Berenj, Bengal, M202 and Saber also showed an *indica* introgression in the region. This example serves as a positive control and demonstrates the tremendous power of introgression mapping in rice, even with only 1311 SNPs. Furthermore, the present study provided greater resolution than the *Oryza*SNP study in tracing the origin of the *sd1* introgression to a specific subpopulation. Despite the higher SNP density, the study by McNally et al. [11] lacked the power to determine whether the original donor of *sd1* belonged to the *indica* or the *aus* subpopulation, while this study clearly identifies it to be of *indica* origin. This illustrates the trade-off in power between the number of SNPs and the number of accessions. In the future, dramatic enhancements in genotyping efficiency will lower the cost of high-resolution genotyping so that gains in resolution will be possible at a fraction of the current cost. This will make it possible to evaluate much larger numbers of SNPs across tens of thousands of accessions, enormously increasing both the power and the resolution of evolutionary analysis.

### *Pi-ta*: an important gene for rice blast resistance

The blast fungus *Magnaporthe oryzae* [50] is a major cause of yield loss in rice, particularly in the drought-prone upland environment where plants suffer from a combination of both water stress and disease. To date, around 60 *R*-genes that provide resistance to specific races of the blast fungus (*Magnaporthe oryzae*) have been mapped on the rice genome [51], [52], and half a dozen have been cloned, including *Pi-ta* [53], [54], *Pi-b* [53], *Pi-9* [55], *Pi-37* [56], *Pi-km* [57] and *Pi-5* [58].

*Pi-ta* is one of the molecularly well-characterized *R*-genes; it encodes a putative cytoplasmic nucleotide binding site (NBS)-type receptor [54] and is located near the centromere at 10.6 Mb on chromosome 12. *Pi-ta* provides complete resistance to two major U.S. pathogen races, IB-49 and IC-17 [59]. Most donors of blast resistance, including *Pi-ta*, are known to be of *indica* origin, but because *tropical japonica* varieties are best adapted to upland growing conditions, breeders have frequently introgressed these resistance genes to enhance the productivity of *tropical japonica* cultivars. In our diversity panel, an extensive segment of *indica* DNA located in the centromeric region on chromosome 12 is found in several *tropical japonica* and admixed accessions (Figure 3), including Kaybonnet, Katy, Spring, Cybonnet, Jambu and Padi Kasalle. Pedigree records indicate that the *Pi-ta* gene in the US varieties can be traced back to the cultivar, Tetep, a Vietnamese *indica* strain that was not included in this study [60], [61].

Because the functional T/G SNP that leads to the change from serine to alanine at the 918^th^ amino acid of the protein [53], [54] was included on the GoldenGate array, it provided a “perfect” marker that could be used to determine which accessions carried the resistance allele at the *Pi-ta* locus. Our genotyping results are 100% concordant with the introgression results and prior knowledge about which varieties carry the *Pi-ta* resistance allele (G-allele), including variety registration records [47], [62]–[64].

### *Waxy* (*Wx*): a gene conferring amylose content in the grain

**Figure 4:**
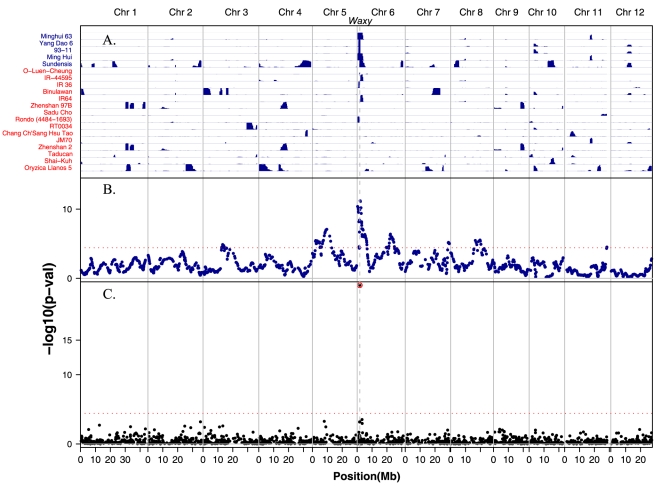
Regions of introgression from *temperate japonica* into *indica* aligned with admixture mapping and association mapping p-values for amylose content. Genome position of SNPs and introgressions indicated across the bottom; vertical grey lines indicate chromosomes; position of *Waxy* gene on chromosome 6 shown as vertical dashed line. (A) Horizontal grey lines indicate accessions; blue-colored regions represent introgressions (defined by ≥5 SNPs) from *temperate japonica* into *indica*; variety names (on left) colored dark blue indicate *indica* accessions carrying a *waxy* allele introgressed from *temperate japonica*; those without *waxy* introgression indicated in red. (B) Admixture mapping p-values for amylose content using the *temperate japonica* component in the *admixed* subpopulation. (C) Association mapping p-values for amylose content in all accessions using mixed model approach. Horizontal dotted lines in both (B) and (C) represent significance values of 0.05 after Bonferroni correction.

The glutinous phenotype of rice is largely controlled by the *Waxy* (*Wx*) gene, located at 1.7 Mb on the short arm of chromosome 6. *Waxy* is a granule-bound starch synthase that is responsible for amylose biosynthesis in the grain [65]. There are two major functional haplotype groups of *Wx* that differentiate the *Indica* and *Japonica* rice varietal groups, with *Wx^a^* found mostly in *Indica* and *Wx^b^* mostly in *Japonica*. The amylose content is significantly lower in the *Wx^b^* haplotype [66]. In our study, the *indica* varieties Ming Hui, Sundensis, IR-44595, 93-11, Yang Dao 6, and Minghui 63 have a *temperate japonica* introgression at the *Wx* locus (Figure 4A). A SNP at the functional G/T mutation in intron 1 that is indicative of the *Wx^b^* haplotype [67] was included on our GoldenGate assay and our genotyping results confirmed that all of the *indica* varieties carrying the introgression marked by the T-allele have significantly lower amylose content (all less than 18.5% with mean = 14.1%) than those carrying the G-allele (all no less than 20% with mean = 23.6%).

### Admixture mapping and association mapping of amylose content and grain length

Admixture mapping is an efficient strategy for associating genotypic and phenotypic variation across parental subpopulations [68]. It makes use of subpopulation differences to identify introgresssed regions resulting from the admixture and has the advantage of requiring fewer markers than association mapping in homogeneous populations. Because the amylose content of *temperate japonica* varieties is typically lower than that of *indica* varieties (Supplemental Figure S3), we were able to use this varietal difference to assess the efficacy of admixture mapping in rice. To do so, we identified the chromosomal regions in *admixed* accessions where increased *temperate japonica* ancestry corresponded to lower amylose content. Specifically, using the *temperate japonica* component estimation in the *admixed* accessions resulting from the introgression analysis above as a predictor, we fitted a linear model for amylose content. The most significant markers fell right near the *Waxy* gene with a p-value of 6.1×10^−12^ (Figure 4B). As a comparison, we also carried out direct association mapping in the whole sample on each SNP using a mixed model approach (See Materials and Methods for more details). The SNPs on the *Waxy* gene have the highest significance, with the functional SNP being the most significant at P<10^−20^ (Figure 4C). Because admixture mapping uses neighboring SNP information to estimate the ancestry component, it gives stronger signal near a causal gene (i.e., *Waxy*) and is often more powerful than association mapping with sparse genotype data, particularly when there are no SNPs in LD with the causal SNP.

**Figure 5:**
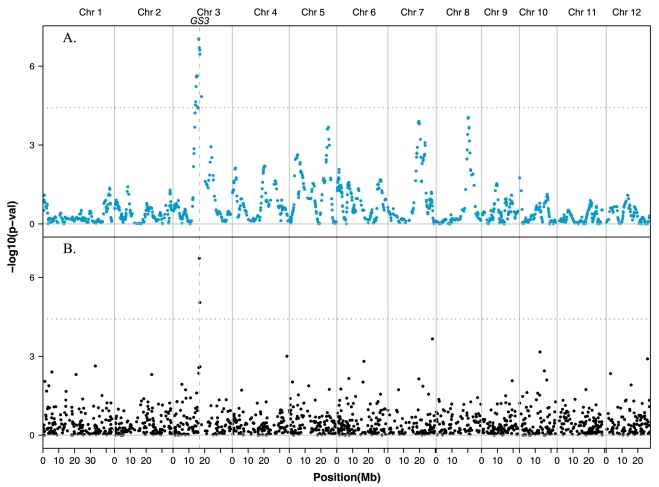
Admixture mapping and association mapping p-values for grain length. (A) Admixture mapping p-values for grain length using the *tropical japonica* component in the admixed subpopulation. (B) Association mapping p-values for grain length in all accessions using the subpopulation component matrix Q (K = 5) as cofactors in the model. Horizontal dotted lines represent significance value of 0.05 after Bonferroni correction. Chromosomes are separated by vertical grey lines; *GS3* gene on chromosome 3 shown as vertical dashed line.

The following provides an example of the power of admixture mapping compared with association analysis in our diversity panel in the absence of a marker in strong LD with the functional SNP target. Grain length is an important component of grain quality in rice [69]. Long grain varieties are common in *indica*, *tropical japonica* and *Group V* varieties while they are rare in *temperate japonica* and *aus* [25] (Supplemental Figure S4). We carried out admixture mapping for grain length using the *tropical japonica* component estimation in the *admixed* accessions as a predictor in the linear model. The most significant SNPs (lowest P = 9.1×10^−8^) are located at 16.7 Mb on chromosome 3 (Figure 5A). This is exactly where the grain size gene *GS3* is located [25], [70]. Association mapping using the mixed model on SNP genotypes in the diversity panel as a whole also showed the highest significance in the region containing the *GS3* gene (P∼2×10^−7^) (Figure 5B). When the two SNPs located within 240 kb of the *GS3* gene are excluded from the analysis, association mapping fails to detect any significant SNPs associated with grain length, while admixture mapping still finds plenty of significant SNPs. This example highlights the power of combining phenotypic information with information about subpopulation to identify genomic regions that have been the targets of artificial selection over the course of rice domestication and breeding.

## Discussion

We developed a 1536-SNP genome-wide assay using Illumina's GoldenGate technology and used it to genotype 395 diverse *O. sativa* samples. The assay can be reliably used for diversity analysis, mapping genes associated with phenotypes of interest, and for a variety of breeding applications both within and between subpopulations of rice. The assay was developed from a subset of SNPs discovered using Perlegen hybridization technology and served to verify that most of the discovery-SNPs in the high quality MBML-intersect dataset [11] could be easily converted to assays based on single base extension. Other 1536 SNP chips for rice have been developed in Japan (Masahiro Yano, NIAS, personal communication) and as part of the “HAPLORYZA” project in the Generation Challenge Programme (http://www.generationcp.org/research.php?da = 0897830). Our study provides the highest resolution view of admixture in the rice genome to date and lays the foundation for more effective genetic resource management, more efficient breeding strategies utilizing natural variation, and raises interesting questions about the dynamics of the domestication process in *O. sativa*.

Based on the allele frequencies estimated from our large sample of rice germplasm, researchers can select subsets of verified SNPs that segregate in specific subsets of genetic materials as the basis for targeted assays designed for QTL mapping, pedigree analysis, tests of hybrid or varietal seed purity, marker-assisted selection, etc. In collaboration with colleagues in the USDA and at the International Rice Research Institute (IRRI) in the Philippines, several 384-SNP mini-arrays have recently been designed to provide cost effective strategies for detecting sets of well-distributed polymorphisms within or between particular subpopulations of rice.

Because of the deep subpopulation structure in rice, care must be taken when developing medium- and low-resolution SNP assays to tailor SNP selection to the intended use of the assay and be aware of ascertainment bias that can distort the interpretation of results. Ascertainment bias is particularly problematic for evolutionary studies where phylogenetic relationships are being inferred. In this study, the 14 *Group V* varieties formed a monophyletic group that showed minimal genetic variation. This is likely to be an artifact of SNP ascertainment bias for two reasons. First, the *Oryza*SNP discovery-dataset included only one *Group V* accession [11]. Thus, we were unable to select SNPs that were known to be variable within the *Group V* subpopulation. Secondly, SNPs segregating both within and between *aus*, *indica*, *tropical japonica* and *temperate japonica* were selected for inclusion on the GoldenGate SNP assay. This strategy enabled us to clearly differentiate varieties within these four subpopulations, but it introduced a bias when making evolutionary inferences. To determine how much of a bias, we compared the topology and internal branch lengths of trees generated by the 159,879 SNPs discovered by the *Oryza*SNP project and the 1,311 high-quality SNPs in this study using the 18 accessions shared between the two projects. We document that SNP ascertainment bias affected internal branch lengths but showed no effect on tree topology (Supplementary Figure S5). Most notably, the Illumina assay accentuated differences between the *tropical japonica*, *temperate japonica* and *Group V* subpopulations, as seen by the longer branch lengths, and minimized the *indica – aus* and the *Indica* – *Japonica* differentiation. This also explains the differences in *F_ST_* estimates between the two studies

Introgression analysis provides an efficient and powerful way of localizing chromosomal regions associated with phenotypes of interest, as demonstrated for semi-dwarf stature (*SD1*), blast disease resistance (*Pi-ta*) and amylose content of the grain (*Waxy*). However, we detected no significant excess of introgression in the *GS3* region associated with grain size in any of the subpopulations, despite its detection based on admixture mapping in this study and evidence that the *gs3* allele conferring long grain originated in a *Japonica* ancestor and was introgressed into the *indica* subpopulation [25]. This can be explained by the small size of the introgression (<1 MB) which falls below the resolution of the GoldenGate SNP assay. Similarly, we fail to detect the *Japonica* introgression on chromosome 7 containing the *rc* allele for white pericarp in *indica* varieties [27] and the *Japonica* introgression containing the *badh2* allele for fragrance in *indica* varieties such as Thai Jasmine [71] because of the small size of the introgressed regions. Denser SNP coverage would provide improved resolution for introgression analysis in rice.

The power of admixture and association mapping is dependent on both the density of SNPs and the quality of phenotypic data available for analysis. In this sparse-genotype-based study, admixture mapping was more powerful than association mapping on individual SNP genotypes because it efficiently utilized neighboring markers to infer the ancestry component. Nonetheless, both association and admixture mapping detected clear signal near the *Waxy* and *GS3* genes due to the presence of a few markers located inside or very near to the genes of interest. This demonstrates the promise of our germplasm panel for genome-wide studies to explore the molecular genetic basis of diverse phenotypes in *O. sativa* and highlights the requirement for a denser marker array for detecting linkage disequilibrium between markers and causal loci.

The low SNP density on this array (1 SNP every ∼260 kb) is sub-optimal for genome-wide association mapping in the *indica* and *aus* subpopulations where the average extent of linkage disequilibrium (LD) is estimated to be ≤100 kb [11], [72], [73], but it may be sufficient for association mapping in some elite breeding materials where LD can extend >500 kb due to the small founder population. In future studies, the inclusion of wild relatives of cultivated Asian rice, *O. rufipogon*, will provide valuable opportunities to document the extent and directionality of gene flow between domesticated rice and its wild ancestors and to identify source populations for important domestication alleles.

Through a combination of introgression analysis, admixture mapping and association mapping, we localized several genes important to rice genetics and breeders despite the modest resolution of this SNP assay. This provides a powerful proof of concept for whole genome association mapping in this panel of diverse rice germplasm population and represents an important first step in the development of high throughput genotyping strategies for rice. It also provides geneticists and breeders with a large diversity dataset that can be used immediately to facilitate both gene discovery and the breeding of higher quality and higher yielding varieties of rice.

## Materials and Methods

### Plant Materials

A diverse collection of 395 *O. sativa* accessions including both landraces and elite varieties was used in this study. These accessions were selected to represent the range of geographic and genetic diversity of the species [9], [ 74, G. Eizenga, DBNRRC, Stuttgart, AR, personal communication] and include 18 varieties used for SNP discovery in the *Oryza*SNP dataset [11]. Information about the accessions, including the Genetic Stocks *Oryza*, (GSOR) accession identifier (accessible via the GRIN database; http://www.ars-grin.gov/npgs/), accession name, country of origin, Project_ID, subpopulation identity (based on STRUCTURE analysis of SNP data when K = 5) and phenotypes used is listed in Supplemental Table S1.

### SNP selection and Genotyping

The 1,536 SNP targets were selected from the high quality MBML-intersection data in the *Oryza*SNP project [11]. Raw data was produced by the Illumina GoldenGate assay and allele calling was performed by a novel method developed to handle inbred sample collections as well as to overcome limitations of more traditional clustering-based approaches for genotype calling [75]. Details about SNP selection, genotyping and SNP quality control are described in the Supplemental Text S1, Figure S6, Table S2 and Wright et al [75]. All of the data from this study are publically available at http://www.ricediversity.org/IlluminaSNPrelease/ and in the Diversity module of the Gramene database (http://www.gramene.org).

### Population structure and phylogenetic analysis

The Bayesian cluster estimation of population structure was done using the software STRUCTURE. Ten replicates were performed for each value of K, the number of clusters considered. Each run used a burn-in period of 20,000 iterations followed by 10,000 iterations. The best replicate giving the maximum likelihood were chosen as the final result for each K. Inferred ancestry for each accession when K = 5 are given in Supplemental Table S1. We classified each accession based on its maximum subpopulation component. If the maximum value was less than 80%, it was classified as admixed. The phylogenetic analysis of genotypes was performed using PHYLIP [76]. The neighbor-joining tree was constructed based on the allele-sharing distance.

The *F_ST_* was calculated using the unbiased estimate that corrects for the variance in sample size over subpopulations [77], [78]. Specifically, for a sample with m subpopulations each with sample size n_i_ (i = 1,…,m), denote the frequency of SNP A allele in the ith subpopulation as p_i_, 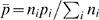 as the sample size weighted average of p_i._ then *F_ST_* can be estimated as follows:
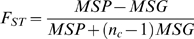
where, MSG and MSP are the observed mean square error within and between subpopulations, respectively.
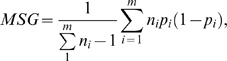


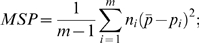


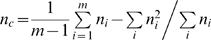
 is the weighted sample size across all subpopulations.

The unbiased estimate correction can produce negative values, which doesn't have any biological meaning. Thus, any negative value was set to 0.

### Introgression analysis

A Bayesian analysis of subpopulation origin along the genome was carried out in *STRUCTURE* assuming *K* = 5 using a site-by-site linkage model without any prior subpopulation assignment. Ten replicate runs were performed. Each run used a burn-in period of 40,000 iterations including 20,000 iterations of initial burn-in with the admixture model for best mixing properties, which was then followed by 20,000 iterations. The best replicate giving the maximum likelihood was chosen as the final result. For each accession, we obtained estimates of the five ancestral components (sum up to 1) for each SNP. The ancestral component of each accession that was not the same as its subpopulation assignment was considered as an introgression from the corresponding subpopulation. For example, for an *indica* accession whose genome is mostly *indica*, those SNPs that showed significant *tropical japonica* ancestry were identified as an introgression from *tropical japonica*. The average introgression level in each subpopulation for a specific ancestral origin at a locus is simply measured as the mean value of the corresponding ancestral components for all accessions in the subpopulation at the locus.

### Admixture mapping and association mapping

The phenotypes of amylose content and grain length were taken as the average of at least 2 reps of each accession measured. The ancestry subpopulation components (Q matrix) were estimated from STRUCTURE using *K* = 5. In admixture mapping, a simple linear regression model was fit on the ancestry component in the admixed subpopulation. Amylose content was regressed on the *temperate japonica* component and grain length was regressed on the *tropical japonica* component, respectively. Association mapping was done using a mixed model with SNP and Q matrix as fixed effects and the estimated genetic relatedness between individuals as the random effect [29], [79].

## Supporting Information

Text S1 SNP selection and genotyping algorithms.
(0.04 MB DOC)

Figure S1 Fst between subpopulation *indica* (IND) and *japonica* (TEJ + TRJ).
(0.48 MB PDF)

Figure S2 Comparison of Introgression from IND (*indica*) into TEJ (*temperate japonica*) and TRJ (*tropical japonica*).
(0.00 MB PDF)

Figure S3 Phenotypic distribution of amylose content in different subpopulations as defined in Supplemental Table S1.
(0.19 MB PDF)

Figure S4 Phenotypic distribution of grain length in different subpopulations as defined in Supplemental Table S1. Grain length is measured as the hulled seed length.
(0.18 MB PDF)

Figure S5 Phylogenetic trees for the common accessions in the GoldenGate and *Oryza*SNP datasets. Both trees are constructed as the neighbor joining tree using the allele-sharing distance matrix.
(0.09 MB PDF)

Figure S6 SNP distribution along the genome. There are 3 rows for each chromosome. From bottom row to top for each chromosome: black bars = *Oryza*SNP MBML-intersect set; red bars = 1536 SNPs on the GoldenGate array; and blue bars represent the 1311 successful SNPs.
(5.48 MB PDF)

Table S1 Sample information and subpopulation assignment when K = 5 and phenotypic information.
(0.11 MB XLS)

Table S2 SNP marker information.
(0.38 MB XLS)
