# Supplementary material for: Genomic Diversity and Introgression in O. sativa Reveal the Impact of Domestication and Breeding on the Rice Genome
Source: PLoS One. 2010 May 24;5(5):e10780. doi: 10.1371/journal.pone.0010780 (PMC2875394; doi:10.1371/journal.pone.0010780)
Supplement: Text S1 — SNP selection and genotyping algorithms. (0.04 MB DOC) [file pone.0010780.s001.doc]

**Supplemental Text 1. SNP selection and genotyping algorithms**

## Selection of SNPs for the Illumina GoldenGate Assay

We selected 1,536 SNPs from the high quality MBML-intersection dataset described by (McNally et al. 2009) that consisted of 159,879 SNPs (false positive rate = 2.8%). During the first stage, we obtained a designability score estimate for a large number of SNPs using the Illumina design tool. For those that had an r^2^ value of 1.0 with other SNPs within 500kb, we prioritized those with high frequency scores in the *tropical japonica, temperate japonica, indica* and/or *aus* subpopulations. To avoid large gaps between SNPs, we included at least one SNP for every 100kb window in the genome, with priority on the SNP with the best frequency score. This initially gave us ~11,000 SNPs. Using 100bp of flanking sequence on either side of the target SNPs (extracted from the Nipponbare genome sequence), we then obtained quality scores from Illumina and discarded SNPs with a SNP_Score <0.6 and Designability Rank <1. SNPs that scored well using the design tool were again thinned, keeping those with high frequency in the target subpopulations. Our final selection identified a set of 1,536 well-distributed SNPs (Supplemental Fig. S6). Information about marker position, including 60 bp of flanking sequence on either side of the target SNPs, and allele information are found in Supplemental Table S2.

## Genotyping

The automated calling of genotypes from raw data produced by the Illumina GoldenGate Assay was performed by a novel method developed to handle inbred sample collections as well as to overcome the limitations of more traditional clustering based approaches. The default allele-calling software provided by Illumina (BeadStudio), as well as many third party genotype calling methods, have been developed for outcrossing species and assume the existence of three distinct clusters in the data, as predicted for populations at Hardy Weinberger equilibrium. If only two clusters are observed, one is presumed to be the heterozygote class. In our case, the use of mostly inbred materials resulted in a paucity of heterozygote genotypes and in many cases, no heterozygotes at all. Yet BeadStudio tended to mislabel one of the homozygous clusters, identifying it as either a heterozygote, or dividing one homozygous cluster into two clusters in order to obtain the expected 3-cluster outcome. To obtain accurate and automated genotype calls, we designed our own model-based genotype calling algorithm that does not depend on *ad-hoc* or generalized clustering methods and can accept *a priori* specified inbreeding coefficients. This allows the method to make adjustments depending on the expectation of heterozygosity. Our method also provides *a posteriori* quality scores on a per-SNP basis so that the reliability of specific SNPs can be evaluated. The method is described in detail in another publication (Wright et al., 2010).

**SNP quality evaluation**

From the original 1,536 SNPs on the array, 1,311 had high quality scores and were determined to work reliably – i.e. allowed clear clustering of genotypes in our dataset with >1% minor allele frequency. The working SNPs on this assay are well distributed along the genome, as illustrated in Supplemental Figure S6. We used only the 1,311 high quality SNPs for all of our analyses. As controls for genotyping, we used DNA samples from two inbred varieties, Nipponbare and 93-11, which have fully sequenced genomes, and a pseudo-F1 (Nipponbare: 93-11 mixed at 1:1 ratio) on each 96-sample plate. The concordance rate of the same sample across plates was >99%, all Nipponbare and 93-11 samples have genotyping accuracies > 99% and most have a call rate of ~97% (Wright et al., 2010). The F1 and pseudo-F1s also have accuracies of >99% and call rates >95%.

As an additional control, we also genotyped 18 of the accessions used for SNP discovery in the OryzaSNP panel (McNally et al., 2009), and the concordance between the two datasets is consistent with the 2.8% error rate estimated for the OryzaSNP data (Wright et al., 2010). A few accessions had slightly lower concordance rates that are predicted to be caused by the different sources of DNA used in the two projects.
