# Supplementary figures and images for: Genomic Diversity and Introgression in O. sativa Reveal the Impact of Domestication and Breeding on the Rice Genome
Source: PLoS One. 2010 May 24;5(5):e10780. doi: 10.1371/journal.pone.0010780 (PMC2875394; doi:10.1371/journal.pone.0010780)

# Fst between Indica and Japonica

Chr

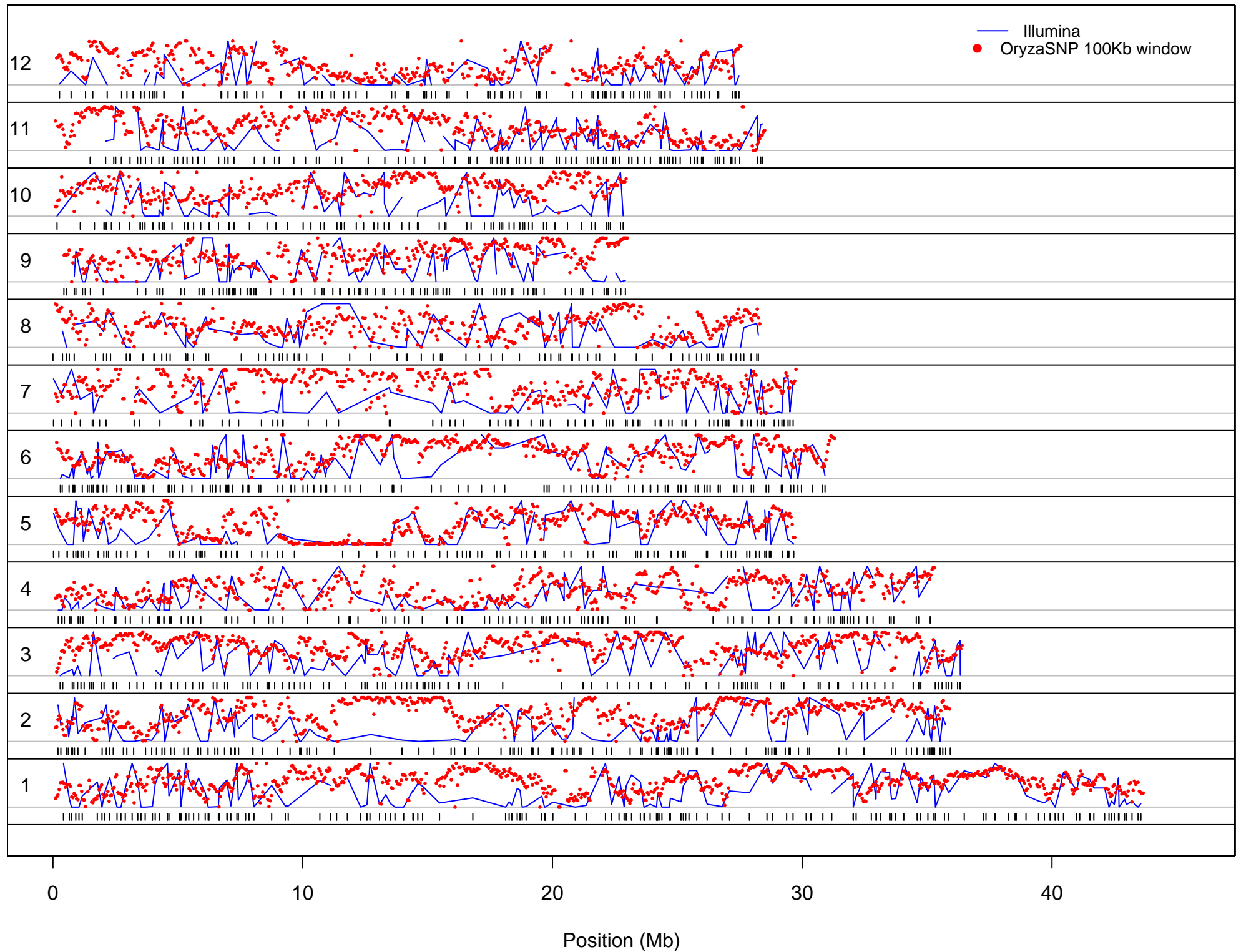

Supplement: Figure S1 — Fst between subpopulation indica (IND) and japonica (TEJ + TRJ). (0.48 MB PDF) [file pone.0010780.s002.pdf]

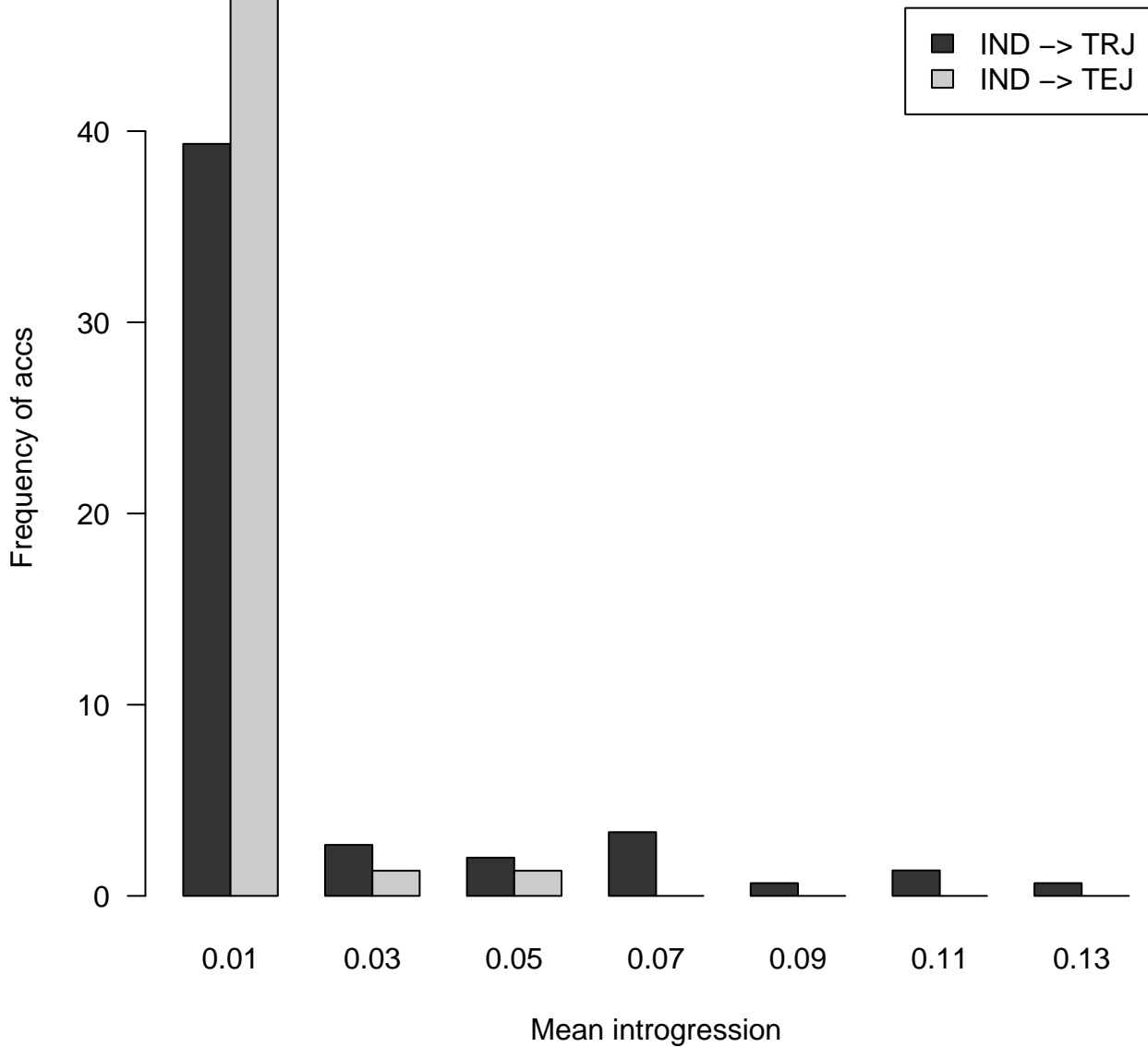

Supplement: Figure S2 — Comparison of Introgression from IND (indica) into TEJ (temperate japonica) and TRJ (tropical japonica). (0.00 MB PDF) [file pone.0010780.s003.pdf]

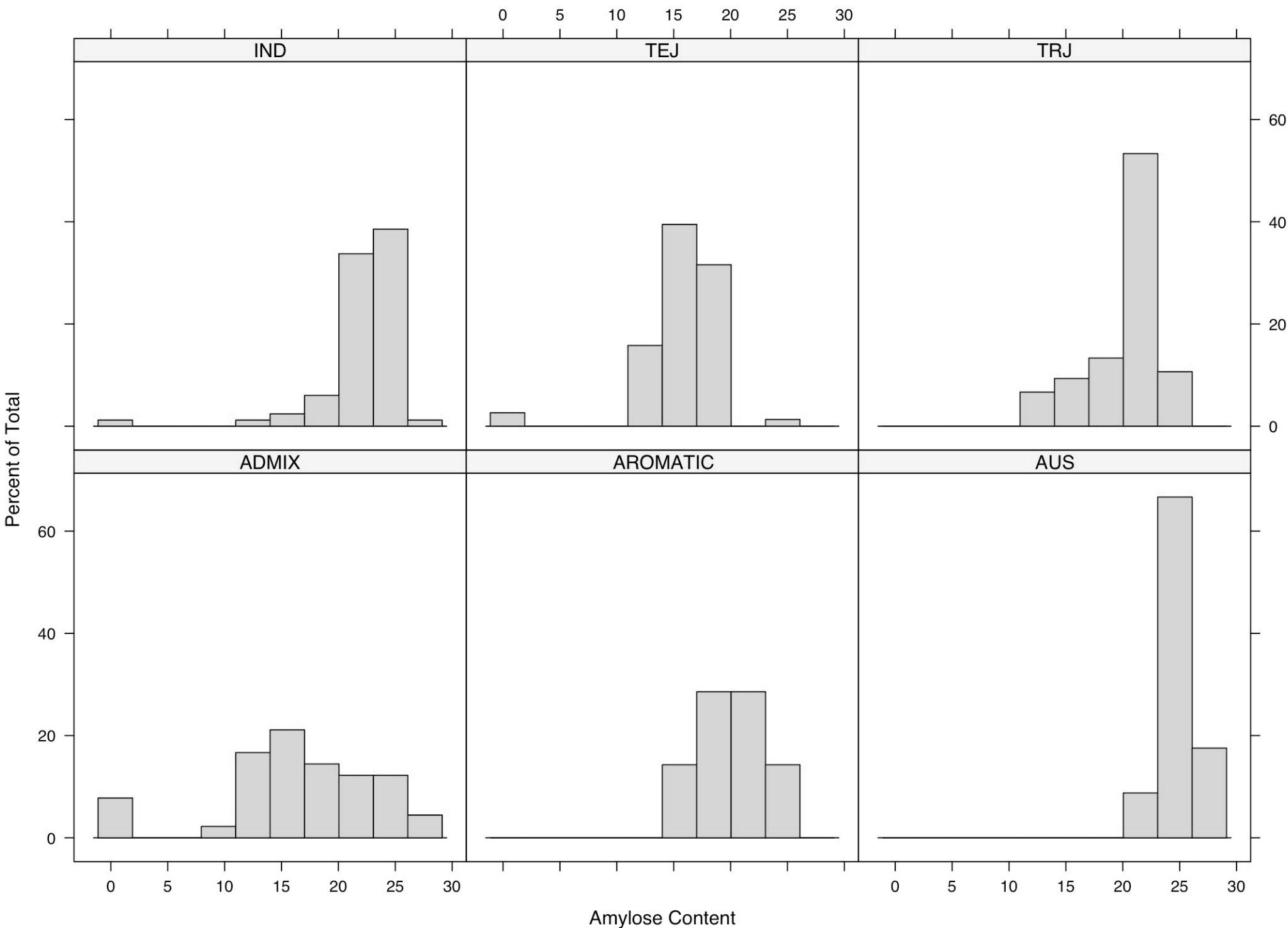

Supplement: Figure S3 — Phenotypic distribution of amylose content in different subpopulations as defined in Supplemental Table S1. (0.19 MB PDF) [file pone.0010780.s004.pdf]

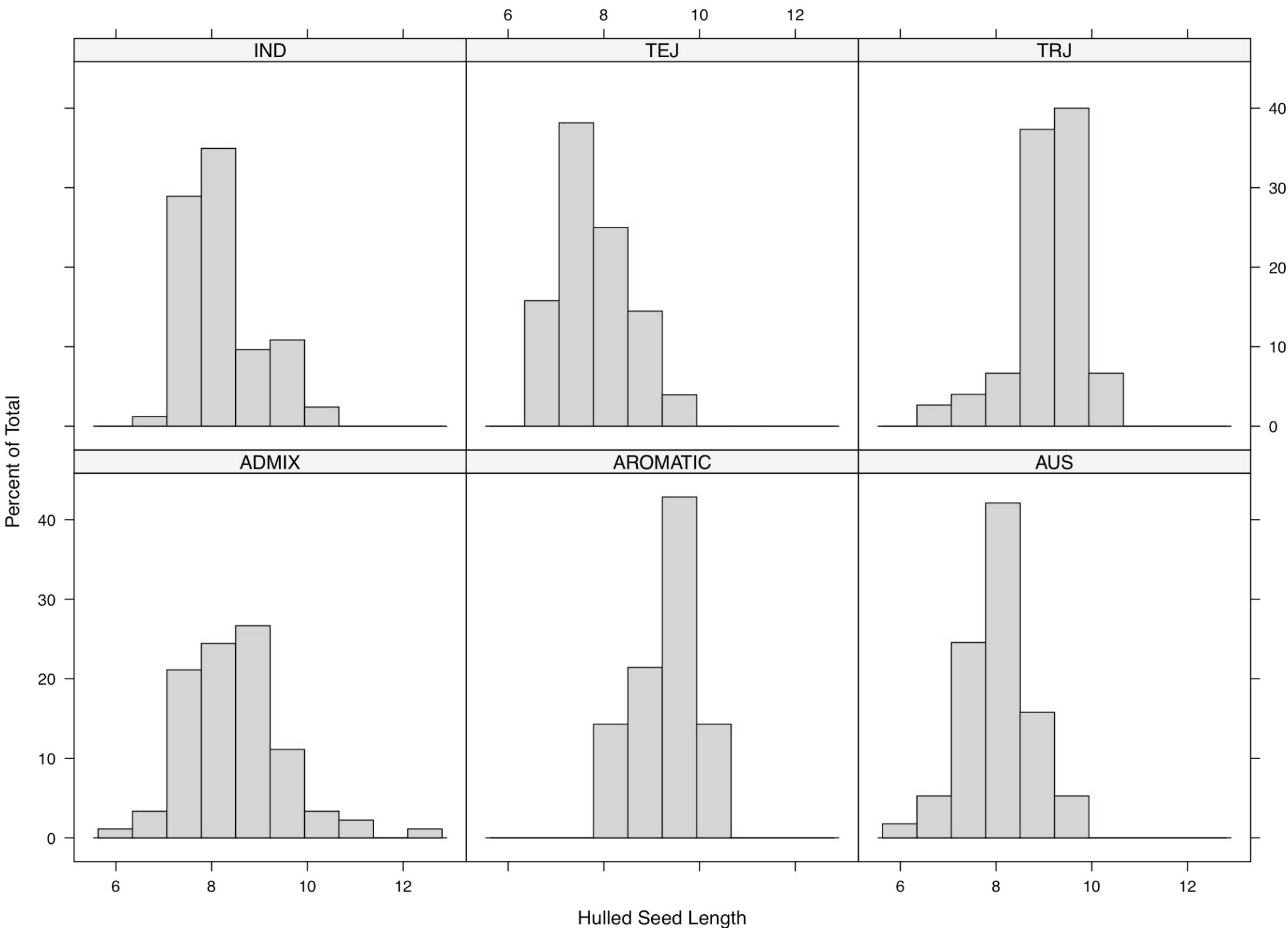

Supplement: Figure S4 — Phenotypic distribution of grain length in different subpopulations as defined in Supplemental Table S1. Grain length is measured as the hulled seed length. (0.18 MB PDF) [file pone.0010780.s005.pdf]

OryzaSNPset

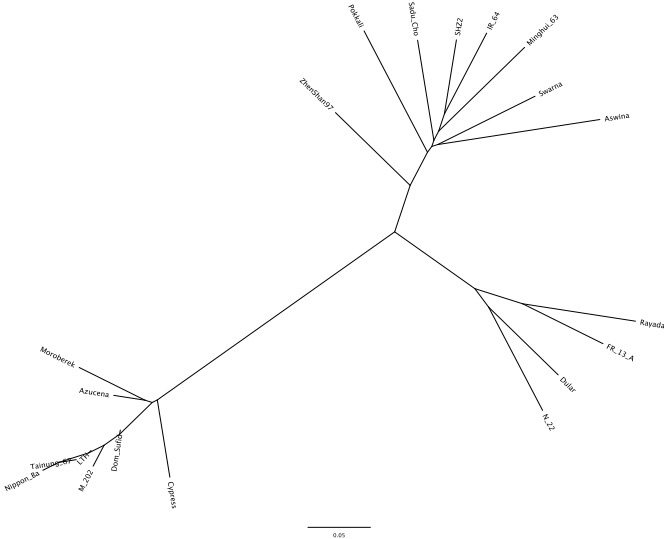

Illumina SNPs

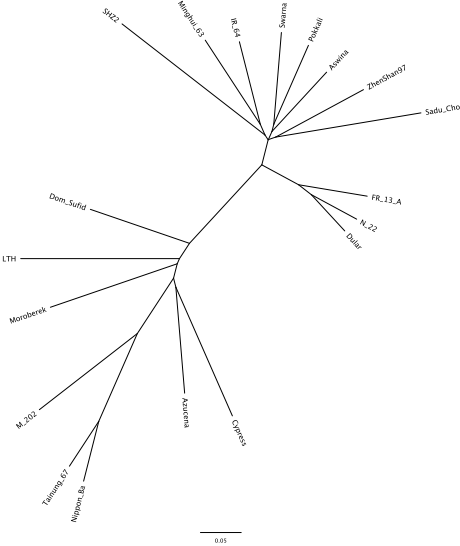

Supplement: Figure S5 — Phylogenetic trees for the common accessions in the GoldenGate and OryzaSNP datasets. Both trees are constructed as the neighbor joining tree using the allele-sharing distance matrix. (0.09 MB PDF) [file pone.0010780.s006.pdf]

SNP positions

Chr

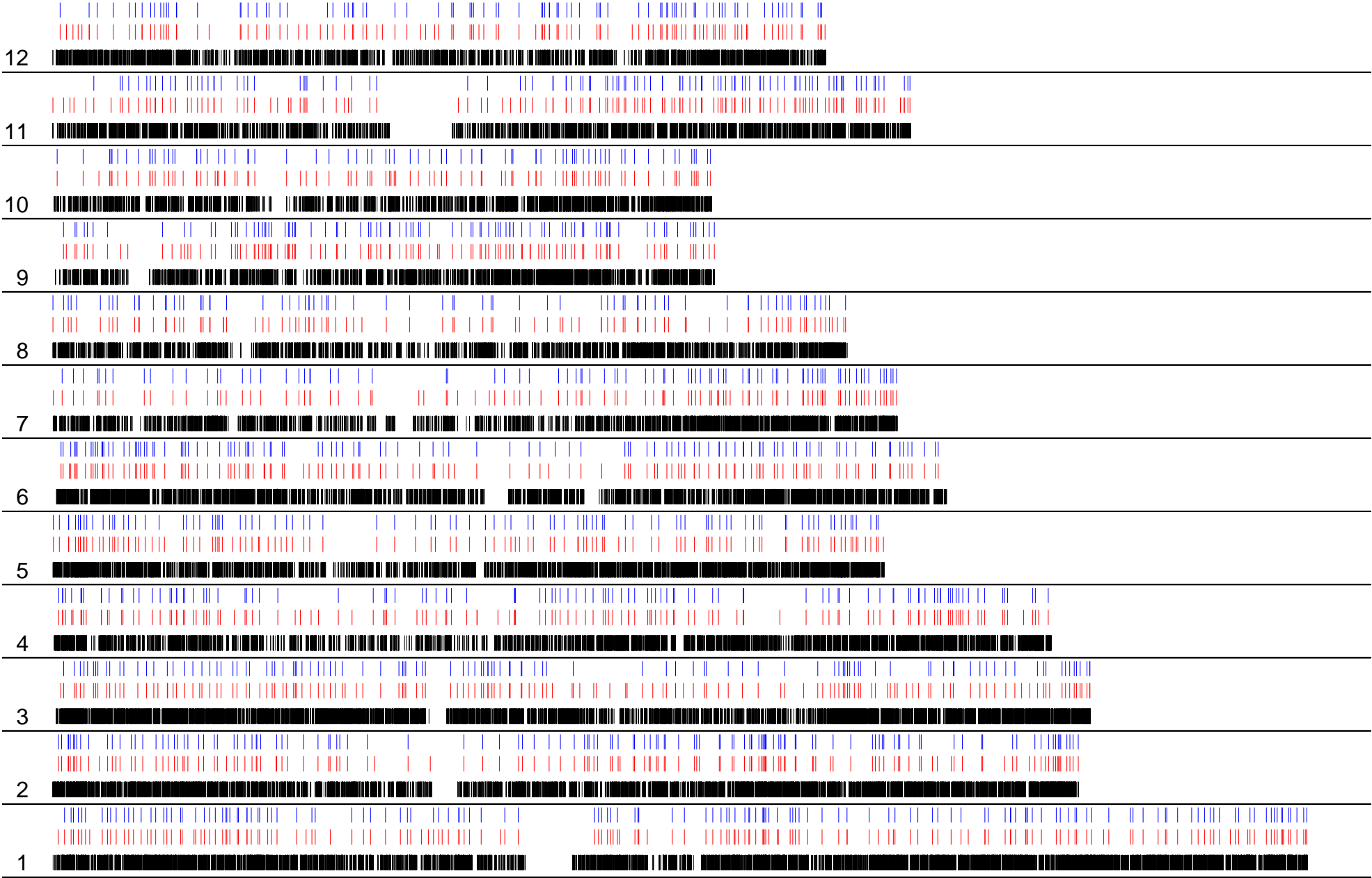

Supplement: Figure S6 — SNP distribution along the genome. There are 3 rows for each chromosome. From bottom row to top for each chromosome: black bars = OryzaSNP MBML-intersect set; red bars = 1536 SNPs on the GoldenGate array; and blue bars represent the 1311 successful SNPs. (5.48 MB PDF) [file pone.0010780.s007.pdf]
